# Phase II study of an oxaliplatin-based regimen for relapsed colon cancer patients treated with oxaliplatin-based adjuvant chemotherapy (INSPIRE study)

**DOI:** 10.1007/s00280-021-04232-2

**Published:** 2021-02-08

**Authors:** Keiichiro Ishibashi, Toru Aoyama, Masahito Kotaka, Hironaga Satake, Yasushi Tsuji, Masato Kataoka, Masato Nakamura, Naoki Nagata, Junichi Sakamoto, Koji Oba, Hideyuki Mishima

**Affiliations:** 1 Department of Digestive Tract and General Surgery Saitama Medical Center, Saitama Medical University, Saitama, Japan; 2 Department of Surgery, Yokohama City University, 3-9 Fukuura, Kanazawa-ku, Yokohama, 2360004 Japan; 3 Department of Gastrointestinal Cancer Center, Sano Hospital, Kobe, Japan; 4 Cancer Treatment Center, Kansai Medical University Hospital, Hirakata, Japan; 5 Department of Medical Oncology, KKR Tonan Hospital, Sapporo, Japan; 6 Department of Surgery, National Hospital Organization Nagoya Medical Center, Nagoya, Japan; 7 Department of Chemotherapy Comprehensive Cancer Center, Aizawa Hospital, Nagano, Japan; 8 Kitakyushu General Hospital, Kitakyushu, Japan; 9 Tokai Central Hospital, Kakamigahara, Japan; 10 Department of Biostatistics, School of Public Health, Graduate School of Medicine, The University of Tokyo, Tokyo, Japan; 11 Cancer Center, Aichi Medical University, Nagakute, Japan

**Keywords:** Oxaliplatin, Re-introduction, Colon cancer

## Abstract

**Background:**

The aim of this study was to evaluate the efficacy and safety of first-line chemotherapy with re-introduction of oxaliplatin (OX) more than 6 months after adjuvant chemotherapy including OX.

**Methods:**

Stage II/III colon cancer patients with neuropathies of grade ≤ 1 who relapsed more than 6 months after adjuvant chemotherapy including OX were considered eligible. Eligible patients were treated with 5-fluorouracil, *l*-leucovorin and OX plus molecularly targeted agents or capecitabine and OX plus bevacizumab (BV) or S-1 and OX plus BV. The primary endpoint was the progression-free survival (PFS), and the secondary endpoints were the overall survival (OS), response rate (RR) and toxicity.

**Results:**

A total of 50 patients were enrolled between September 2013 and May 2019. Twelve patients received 5-fluorouracil, *l*-leucovorin and OX (FOLFOX) plus BV, 21 patients received capecitabine and OX plus BV, 10 patients received S-1 and OX plus BV and 7 patients received FOLFOX plus cetuximab or panitumumab. The median PFS was 11.5 months (95% confidence interval [CI] 8.3–16.0), the median OS was 45.4 months (95% CI 37.4–NA), and the RR was 56.0% (95% CI 42.3–68.8). Adverse events of grade ≥ 3 that occurred in ≥ 5% of cases were neutropenia in 6 patients (12%), peripheral sensory neuropathy in 5 patients (10%), diarrhea in 4 patients (8%), hypertension in 4 patients (8%), anorexia in 3 patients (6%) and allergic reactions in 3 patients (6%).

**Conclusions:**

First-line chemotherapy with re-introduction of OX more than 6 months after adjuvant chemotherapy including OX can be used safely with expected efficacy for relapsed colon cancer patients.

## Introduction

Colon cancer is the third-most commonly diagnosed cancer, with an estimated 1,400,000 new cases and 700,000 deaths globally each year [1]. Chemotherapy is an essential method of colon cancer treatment [2–4]. Among the various chemotherapy agents, oxaliplatin (OX) is one of the most substantial key agents for colon cancer treatment in both adjuvant and unresectable-metastatic disease settings.

Thus far, three pivotal studies have shown that OX-based adjuvant chemotherapy, such as infusional 5-fluorouracil, *l-*leucovorin and OX (FOLFOX) or capecitabine and OX (CAPOX), for colon cancer significantly improved both the overall survival (OS) and disease-free survival [5–7]. OX-based adjuvant chemotherapy for colon cancer has been widely accepted and performed in clinical practice, and FOLFOX and CAPOX are also widely used in both the first and the second lines for metastatic colon cancer [8–10]. However, there is little supporting evidence available, and few studies have evaluated the efficacy and safety of OX re-introduction as the first-line treatment for relapsed colon cancer after OX-based adjuvant chemotherapy [11, 12]. To establish the optimal use of OX for colon cancer treatment, it is necessary to investigate the clinical benefit of OX re-introduction as the first-line treatment for relapsed disease after OX-based adjuvant chemotherapy.

The present study evaluated the efficacy and safety of first-line chemotherapy with re-introduction of OX more than 6 months after the completion of adjuvant chemotherapy with an OX-containing regimen.

## Patients and methods

### Study design

This study was a single-arm, multicenter, phase II study to evaluate the efficacy and safety of physician’s choice OX-based regimen for colon cancer patients with neuropathies of grade < 1 who relapsed more than 6 months after OX-based adjuvant chemotherapy.

Study data and informed consent were obtained in accordance with the Declaration of Helsinki. The Certified Clinical Research Review Board of Aichi Medical University Hospital approved this study protocol. This trial was registered with the UMIN Clinical Trials Registry as UMIN 000011348 https://upload.umin.ac.jp/cgi-open-bin/ctr/ctr_view.cgi?recptno=R000013300. This trial was registered with the Japan Registry of Clinical Trials as jRCTs041180118. https://jrct.niph.go.jp/latest-detail/jRCTs041180118; all patients were given a written explanation and provided their written informed consent before participating.

### Inclusion and exclusion criteria

Tumors were staged according to the UICC version 7 [13]. The inclusion criteria were as follows: (1) stage II/III colon cancer with neuropathies of grade ≤ 1 who relapsed more than 6 months after adjuvant chemotherapy including OX; (2) performance status of 0–1; (3) ≧ 20 years of age; (4) presence of at least one measurable lesion using the Response Evaluation Criteria in Solid Tumors (RECIST) version 1.1; (5) past history of adjuvant chemotherapy including OX with a cumulative dose of more than 300 mg/m^2^; (6) adequate hematologic, liver, and coagulation profiles and normal electrocardiogram findings; and (7) consent given to participate in this clinical study. The exclusion criteria were as follows: (1) serious coexisting morbidities; (2) active synchronous or metachronous malignant disease; (3) pregnant or lactating; (4) not considered suitable for participation for any other reason.

### Treatment methods

Eligible patients were treated with infusional FOLFOX plus molecularly targeted agents or CAPOX plus bevacizumab (BV) or S-1 and OX (SOX) plus BV. Selection of OX-based regimen was decided by the attending physician at registration of each patient. FOLFOX was administered as a 2-h OX 85 mg/m^2^ infusion on day 1 in tandem with a 2-h *l*-leucovorin 200 mg/m^2^ infusion on day 1 and 5-FU as a 400-mg/m^2^ bolus followed by a 46-h 2400 mg/m^2^ infusion on days 1 to 3, every 2 weeks. In addition, BV (5 mg/kg on day 1) or cetuximab (400 mg/m^2^ as the initial dose and 250 mg/m^2^ as the subsequent doses on days 1 and 8) or panitumumab (6 mg/kg on day 1) was combined with FOLFOX. CAPOX plus BV was administered as intravenous OX 130 mg/m^2^ on day 1, oral capecitabine 1000 mg/m^2^ twice daily from the evening of day 1 to the morning of day 15 and BV 7.5 mg/kg on day 1, every 3 weeks. SOX plus BV was administered as intravenous OX 130 mg/m^2^ on day 1, oral S-1 40 mg/m^2^ twice daily from the evening of day 1 to the morning of day 15 and BV 7.5 mg/kg on day 1, every 3 weeks.

### Endpoints

The primary endpoint was the progression-free survival (PFS). The secondary endpoints were the OS, response rate (RR) and the safety of the combination therapy. Radiographic image studies were performed every eight weeks. The RR was evaluated by the RECIST 1.1 criteria [14]. All adverse events recorded were graded according to the Common Terminology Criteria for Adverse Events of the National Cancer Institute (CTCAE) version 4.0 [15]. The PFS was defined as the period between the day of registration and progression or death, whichever came first. Patients were censored at the last point when no progression was confirmed if the patients did not experience any event associated with the PFS. The OS was defined as the period between the day of registration and death. The data of patients who had not experienced an event were censored at the date of the final observation.

### Statistical analyses

We set the threshold median PFS at 7 months and the expected median PFS at 10.5 months based on the results of a previous study [16–19]. Given a 2-sided alpha of 0.05 and statistical power of 80% with about 10% ineligible or dropout patients, we set 50 patients as the target sample size in this study.

The analytical population for efficacy was defined as all eligible patients, and that of safety was defined as all eligible patients who received treatment at least once. In the present study, disease control rate (DCR) was defined as the percentage of complete response, partial response, and stable disease in full set analysis. The PFS and OS curves were calculated using the Kaplan–Meier method, and the 95% confidence interval (CI) was estimated using the Brookmeyer and Crowley method with log–log transformation. All analyses were implemented by SAS 9.4, SAS/STAT 14.2 (SAS Institute, Cary, NC,USA).

#### Results

### Patients’ background characteristics

From September 2013 to May 2019, 50 patients were registered from 21 institutions. The intension-to-treat analysis and safety analysis were carried out on those 50 patients.

Table 1 shows the patients’ background characteristics. Twenty-eight patients were male, and 22 were female, with a median age of 69.5 years (range 27–82 years). The time until recurrence from the completion of adjuvant therapy was 6–12 months in 16 patients, 12–24 months in 15 patients and more than 24 months in 19 patients. The median total dose of OX for adjuvant chemotherapy were 1136 (470–1904) mg/body. The most common metastatic site was the lung (22 patients, 44%), lymph node (19 patients, 38%), peritoneal metastasis (13 patients, 26%) and liver (11 patients, 22%). The median follow-up was 34.3 months (range 20.8–63.7 months). Twelve patients received FOLFOX plus BV, 21 patients received CAPOX plus BV, 10 patients received SOX plus BV, and 7 patients received FOLFOX plus cetuximab or panitumumab.

**Table 1:** Patient characteristics

Characteristics	No. of patients	(%)
Gender		
Male	28	56.0
Female	22	44.0
Age (years)		
Median	69.5	
Range	27–82	
Performance status (PS)		
0	44	88.0
1	6	12.0
Cancer location		
Colon	29	58.0
Rectum	21	42.0
Previous adjuvant chemotherapy		
FOLFOX	16	32.0
CAPOX	32	64.0
Other	2	4.0
Time from adjuvant chemotherapy		
6 -12 months	16	32.0
12–24 months	15	30.0
More than 24 months	19	38.0
Oxaliplatin free interval		
6–12 months	15	30.0
12–24 months	14	28.0
More than 24 months	21	42.0
Baseline peripheral sensory neuropathy		
0	31	62.0
1	19	38.0
Number of relapse site		
0	0	0
1	32	64.0
> 2	18	36.0

*FOLFOX* infusional 5-fluorouracil, *l*-leucovorin and oxaliplatin, *CAPOX* capecitabine and oxaliplatin, Intention to treat population, *n* = 50

### Efficacy

**Fig. 1:**
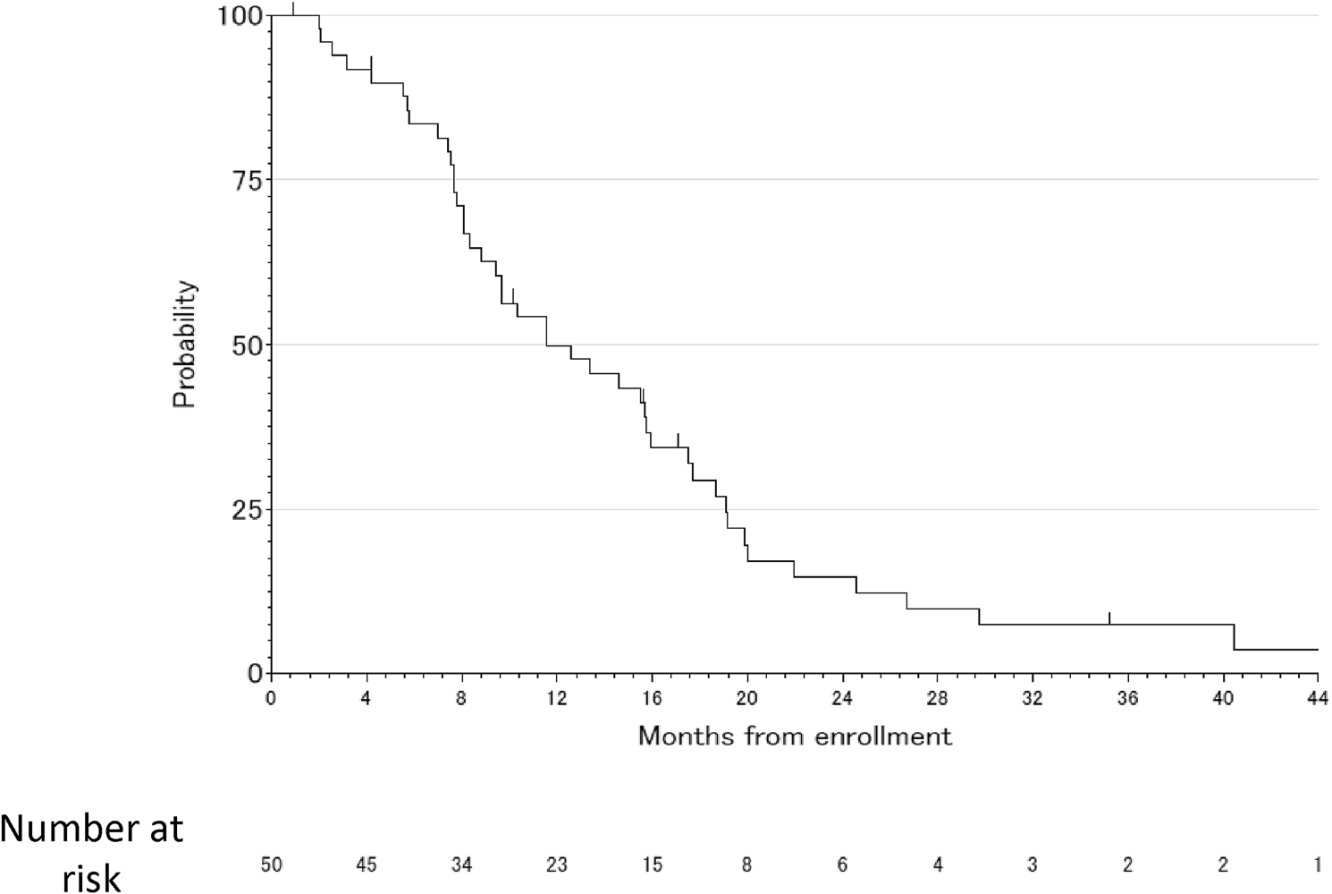
The progression-free survival

**Fig. 2:**
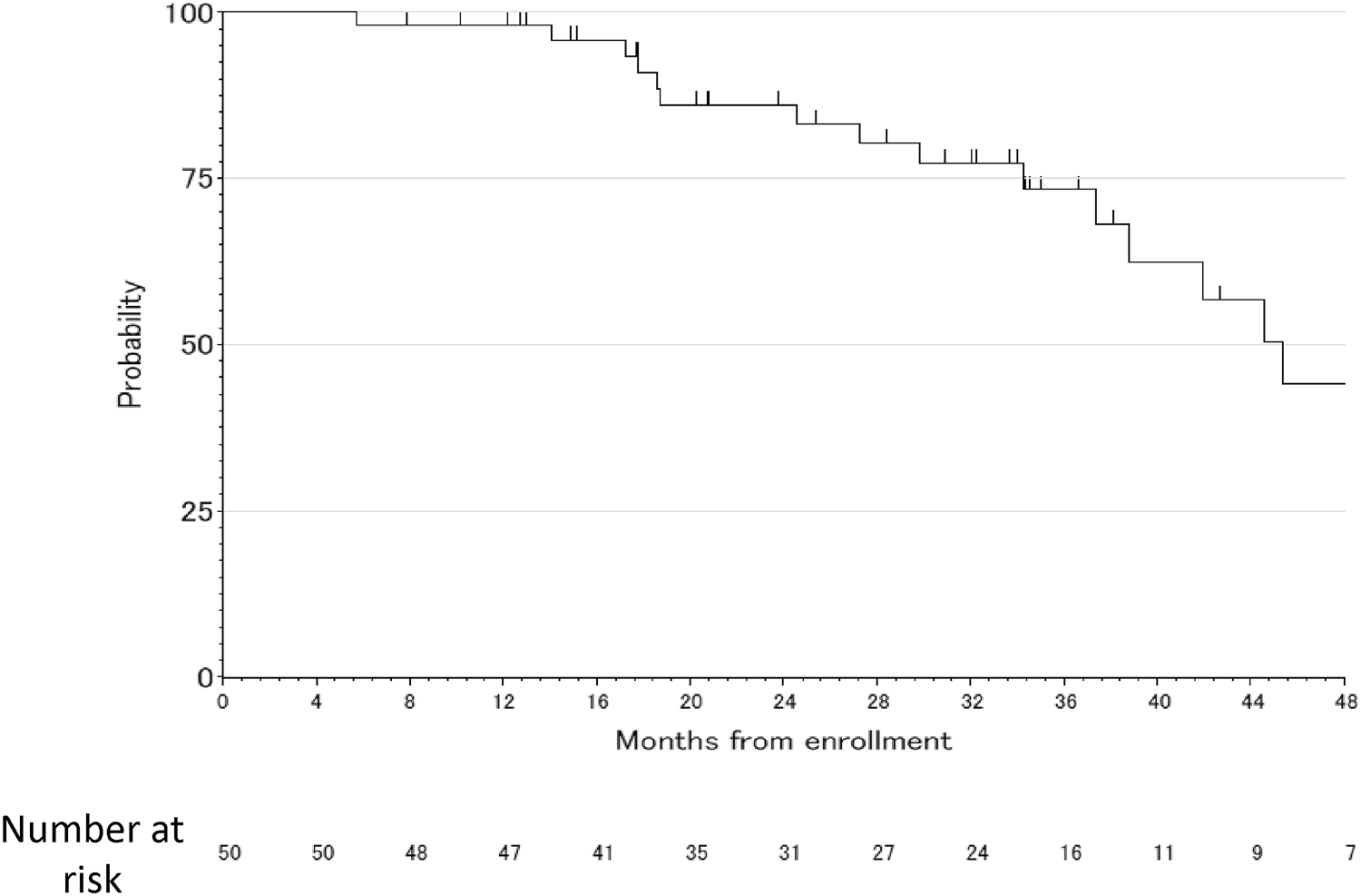
The overall survival

All follow-up data were collected by Dec/2019 and the median follow-up period was 34.3 months. The median PFS was 11.5 months (95% CI 8.3–16.0 months) (Fig. 1). The median PFS among subgroups based on time from the completion of adjuvant chemotherapy (6–12 months/12–24 months/more than 24 months) was comparable [13.0 months (95% CI 7.0–19.2)/11.0 months (95% CI 7.5–19.9)/12.7 months (95% CI 7.8–17.7), respectively]. The median OS was 45.4 months (95% CI 37.4 months–NA) (Fig. 2). The reasons for discontinuing the study treatment included progression of the primary disease in 26 patients (54.2%), adverse events in 5 patients (10.4%) (Platelet count decreased was 2 patients, Urine protein was 1 patient, Neutropenia was 1 patient, Anorexia was 1 patient), discretion of the physician in 4 patients (8.3%), refusal by 6 patients (12.5%) and withdrawal of 3 patients (6.3%) due to confirmation of complete response (CR). Two patients continued the protocol treatment. The median OS among subgroups based on time from the completion of adjuvant chemotherapy (6–12 months/12–24 months/more than 24 months) was comparable [44.6 months (95% CI, 24.6-NA)/45.4 months (95% CI, 27.3-NA)/61.3 months (95% CI, 18.6-NA), respectively]. According to the subgroup analysis for OX-free interval, the median PFS and OS were 13.4 months (95% CI 7.0–19.2) and NA months (95% CI 41.9–NA) respectively for 6–12 months, 10.4 months (95% CI 7.4–19.9) and 37.4 months (95% CI 18.7–NA) respectively for 12–24 months, and 12.1 months (95% CI 7.7–17.5) and 45.4 months (95% CI 29.8–NA) respectively for more than 24 months.

Table 2 shows the efficacy data. The best overall RR was 56.0% (95% CI 42.3–68.8%). The disease control rate (DCR) was 86.0% (95% CI 73.5–93.4%). In the present study, the best overall RR for OX-free interval was 53.3% (8/15) for 6–12 months, 71.4% (10/14) for 12–24 months and 47.6% (10/21) for more than 24 months. Four patients were converted to be resectable and underwent curative resection.

**Table 2:** Efficacy data

Parameter	Number of patients	(%)
Best overall response rate		
Complete response (CR)	5	10.0
Partial response (PR)	23	46.0
Stable disease (SD)	15	30.0
Progressive disease (PD)	4	8.0
Not assessable	3	6.0
Best overall response rate (CR + PR)	28	56.0
95% CI		42.3–68.8
Disease control rate (CR + PR + SD)	43	86.0
95% CI		73.5–93.4

### Treatment compliance and safety

Table 3 shows the treatment exposure. The median total dose of OX was 525 mg/m^2^ (85–1690 mg/m^2^). The median total dose of OX was 348 mg/m^2^ (85–1615 mg/m^2^) for FOLFOX plus BV, 650 mg/m^2^ (130–1645 mg/m^2^) for CAPOX plus BV, 525 mg/m^2^ (260–1690 mg/m^2^) for SOX plus BV and 770 mg/m^2^ (170–1235 mg/m^2^) for FOLFOX plus cetuximab or panitumumab. The median course of the study treatment was 14 cycles in FOLFOX plus BV, 10 cycles in CAPOX plus BV, 6 cycles in SOX plus BV and 15 cycles in FOLFOX plus cetuximab or panitumumab.

**Table 3:** Treatment exposure of oxaliplatin

Oxaliplatin total dose (mg/m^2^)	Regimen			
	FOLFOX plus BV	CAPOX plus BV	SOX plus BV	FOLFOX plus Cmab or Pmab
*n*	12	21	10	7
Mean	540	701	718	710
Std	475	392	501	406
Min	85	130	260	170
Median	348	650	525	770
Max	1615	1645	1690	1235

*FOLFOX* infusional 5-fluorouracil, *l*-leucovorin and oxaliplatin, *CAPOX* capecitabine and oxaliplatin, *SOX* S-1 and oxaliplatin, *BV* Bevacizumab, *Cmab* Cetuximab, *Pmab* Panitumumab

Adverse events (AEs) of any grade were observed in 88.0% (44/50 patients) of patients**.** Table 4 shows the details of the AEs. Adverse events of grade 3 that occurred in ≥ 5% of cases were neutropenia in 6 patients (12%), peripheral sensory neuropathy in 5 patients (10%), diarrhea in 4 patients (8%), hypertension in 4 patients (8%), anorexia in 3 patients (6%) and allergic reactions in 3 patients (6%). There was no case of grade 4 adverse event or treatment-related death.

**Table 4:** Relevant adverse events occurring in ≥ 10% of patients (highest grade per patients)

Adverse event	Grade 3/4		All Grade	
	Number of patients	(%)	Number of patients	(%)
Hematological				
Leukopenia	0	0	25	50.0
Neutropenia	6	12.0	26	52.0
Anemia	0	0	26	52.0
Thrombocytopenia	0	0	28	56.0
No hematological				
ALP increased	1	2.0	20	40.0
Blood bilirubin increased	0	0	21	42.0
Creatine increased	0	0	11	22.0
Peripheral sensory neuropathy	5	10.0	45	90.0
Peripheral motor neuropathy	2	4.0	17	34.0
Stomatitis	1	2.0	24	48.0
Nausea	2	4.0	29	58.0
Vomiting	1	2.0	10	20.0
Diarrhea	4	8.0	20	40.0
Rash	1	2.0	20	40.0
Paronychia	1	2.0	8	16.0
Anorexia	3	6.0	36	72.0
Fatigue	0	0	35	70.0
Allergic reaction	3	6.0	11	22.0
Hand foot syndrome	0	0	27	54.0
Hypertension	4	8.0	18	36.0
Hemorrhage	0	0	8	16.0

## Discussion

The present study evaluated the efficacy and safety of first-line chemotherapy with re-introduction of OX more than six months after the completion of adjuvant chemotherapy including OX. Our findings suggested that first-line chemotherapy with re-introduction of OX more than 6 months after adjuvant chemotherapy including OX could be used safely with the expected efficacy for relapsed colon cancer patients. Therefore, the re-introduction of OX treatment is a viable option for relapsed colon cancer patients who have already been treated with OX-based adjuvant chemotherapy.

The present study showed that the median PFS was 11.5 months for first-line chemotherapy with re-introduction of OX after adjuvant chemotherapy including OX for colon cancer. In the adjuvant setting, very recently, Kotaka et al. showed the similar results. They evaluate the efficacy of reintroducing FOLFOX or CAPOX with or without BV in relapsed 31 colorectal cancer patient who treated OX as adjuvant chemotherapy between October 2012 and October 2016 [12]. They found that median PFS was 10.8 months (95% CI 6.9–18.8 months). In a metastatic setting, recently, a few studies have evaluated the clinical effects of the re-introduction of OX for colorectal cancer after chemotherapy including OX. de Gramont et al. performed an additional analysis of the OPTIMisation of OXaliplatin (OPTIMOX) trial to evaluate the efficacy of OX re-introduction for metastatic colorectal cancer patients. They found that OX re-introduction had an independent and significant impact on the OS (hazard ratio: 0.56, *P* = 0.009) [20]. In addition, Chibaudel et al. evaluated the clinical effects of the re-introduction of OX-based chemotherapy and the OX-free interval (OFI; cut-off value: 6 months) on tumor sensitivity to OX re-introduction in initially unresectable colorectal cancer who received first-line OX-based chemotherapy (OPTIMOX trial) [20–23]. The PFS and OS were 3.0 and 8.8 months in patients with an OFI < 6 months, respectively, and 5.5 and 16.8 months in patients with an OFI ≥ 6 months, respectively. Furthermore, an OFI of ≥ 6 months improved the survival. Given these results, even after chemotherapy including OX, the re-introduction of OX might improve the survival among colon cancer patients, according to the OFI.

In the present study, the best overall RR and DCR were 56.0% and 86.0%, respectively. Although the patient background characteristics and treatment lines have differed among studies, there have been some showing OX sensitivity in patients after OX-based chemotherapy in both adjuvant and metastatic setting. Table 5 summarized the efficacy of the present study and previous studies. In adjuvant setting, Kotaka et al. reported that the RR was 62.1% (95% CI 42.3–79.3) and the DCR was 82.8% (95% CI 64.2–94.2). The RR for oxaliplatin-free interval was 100.0% in months 6–12 and 56.0% after 12 months. In metastatic setting, Suenaga et al. evaluated the re-introduction of OX-based chemotherapy in 33 metastatic colorectal cancer refractory to standard treatment [24]. They reported that the RR was 6.1% (95% CI 2.5–14.7%) and the DCR 66.7% (95% CI 49.7–83.6%). Goebel et al. investigated FOLFOX re-introduction after a break in treatment or following disease progression on another regimen in 29 cases of metastatic colorectal cancer. They found that the re-introduction of OX was feasible and achieved a response or stabilization in 73% of patients [21]. In addition, the OPTIMOX-1 and OPTIMOX-2 studies showed an RR of 19% and DCR of 58%. Interestingly, the OPTIMOX-1 and OPTIMOX-2 studies also showed that the tumor sensitivity differed between the patients with an OFI < 6 months and ≥ 6 months. The respective DCR and RR were 14% and 45% in those with an OFI < 6 months and 22% and 63% in those with an OFI ≥ 6 months. In addition, the progression disease rate sharply decreased from 52% in the patients with an OFI < 6 months to 23% in those with an OFI ≥ 6 months. Although it is difficult to directly compare the results due to differences in the patient profiles and treatments, even after OX-based chemotherapy, the patients still have a potentially OX-sensitive tumor. Furthermore, the tumor sensitivity might also change depending on the OFI.

**Table 5:** Summary of the efficacy of the present study and previous studies

	Present study	REACT study [Ref. 12]	RE-OPEN study [Ref. 24]	Goebel et al. [Ref. 21]
Study population setting	OX-based adjuvant chemotherapy	OX-based adjuvant chemotherapy	OX-based chemotherapy for metastatic setting	OX-based chemotherapy for metastatic setting
Sample size	50 patients	31 patients	33 patients	29 patients
Progression free survival	11.5 months	10.8 months	98 days	18 weeks
Overall survival	45.4 months	28.7 months	300 days	42 weeks
Response rate	56.0%	62.1%	6.1%	21%
Disease control rate	86.0%	82.8%	39.4%	73%

The present study showed that AEs of any grade were observed in 88% of patients. The incidence of both peripheral sensory and motor neuropathies were not increased. According to previous reports, the incidence of the AEs was acceptable. On other hands, in the previous similar reports, the rate of grade 1/2 and 3 allergic reaction was 12.9% and 3.2%, respectively [12]. The rate of grade 1/2 and 3 allergic reaction of the present study was higher than in the previous study. However, the allergic reaction was not main reason for discontinuation of treatment. Therefore, first-line chemotherapy with re-introduction of OX more than 6 months after adjuvant chemotherapy including OX seems able to be used safely for relapsed colorectal cancer patients.

Several limitations associated with the present study warrant mention. First, there might have been some selection bias. This study was a single-arm, multicenter, phase II study and thus might only have included patients considered suited for OX-based chemotherapy. Second, the optimal OFI was unclear. In the present study, we set the OFI as 6 months according to previous studies. It is unclear whether or not a longer OFI affects the survival and OX sensitivity. However, this issue is a difficult problem to solve, because the early relapse after adjuvant chemotherapy is related to more aggressive tumor. Third, we did not collect the proportion against the expected dose of OX in the adjuvant chemotherapy. Although the median dose of the OX in the present study was similar to previous study; the proportion against the expected dose of OX in the adjuvant chemotherapy was important information for sensitivity in OX re-introduction as the first-line treatment after OX-based adjuvant chemotherapy. Considering these, the further study will clarify these issues.

In conclusion, first-line chemotherapy with re-introduction of OX more than 6 months after completion of adjuvant chemotherapy that had included OX was able to be used safely with the expected efficacy for relapsed colon cancer patients. The re-introduction of OX treatment appears to be a viable treatment option for relapsed colon cancer patients treated with OX-based adjuvant chemotherapy.
